# A Focused Review of Ras Guanine Nucleotide-Releasing Protein 1 in Immune Cells and Cancer

**DOI:** 10.3390/ijms24021652

**Published:** 2023-01-13

**Authors:** Tu Chun Hsu, Gisele O. L. Rodrigues, Hila Winer, Julie A. Hixon, Wenqing Li, Nadya I. Tarasova, Scott K. Durum

**Affiliations:** 1 Cytokines and Immunity Section, Cancer Innovation Laboratory, National Cancer Institute, National Institutes of Health, Frederick, MD 21702, USA; 2 Comparative Biomedical Scientist Training Program, National Institutes of Health, Bethesda, MD 20892, USA; 3 Veterinary Diagnostic Laboratory, Department of Pathobiology and Diagnostic Investigation, Michigan State University, Lansing, MI 48910, USA; 4 Cancer Innovation Laboratory, Center for Cancer Research, National Cancer Institute, Frederick, MD 21702, USA

**Keywords:** cancer, leukemia, Ras, RasGEF, RasGRP1

## Abstract

Four Ras guanine nucleotide-releasing proteins (RasGRP1 through 4) belong to the family of guanine nucleotide exchange factors (GEFs). RasGRPs catalyze the release of GDP from small GTPases Ras and Rap and facilitate their transition from an inactive GDP-bound to an active GTP-bound state. Thus, they regulate critical cellular responses via many downstream GTPase effectors. Similar to other RasGRPs, the catalytic module of RasGRP1 is composed of the Ras exchange motif (REM) and Cdc25 domain, and the EF hands and C1 domain contribute to its cellular localization and regulation. RasGRP1 can be activated by a diacylglycerol (DAG)-mediated membrane recruitment and protein kinase C (PKC)-mediated phosphorylation. RasGRP1 acts downstream of the T cell receptor (TCR), B cell receptors (BCR), and pre-TCR, and plays an important role in the thymocyte maturation and function of peripheral T cells, B cells, NK cells, mast cells, and neutrophils. The dysregulation of RasGRP1 is known to contribute to numerous disorders that range from autoimmune and inflammatory diseases and schizophrenia to neoplasia. Given its position at the crossroad of cell development, inflammation, and cancer, RASGRP1 has garnered interest from numerous disciplines. In this review, we outline the structure, function, and regulation of RasGRP1 and focus on the existing knowledge of the role of RasGRP1 in leukemia and other cancers.

## 1. Ras Guanine Nucleotide Exchange Factors: Introduction

**Figure 1:**
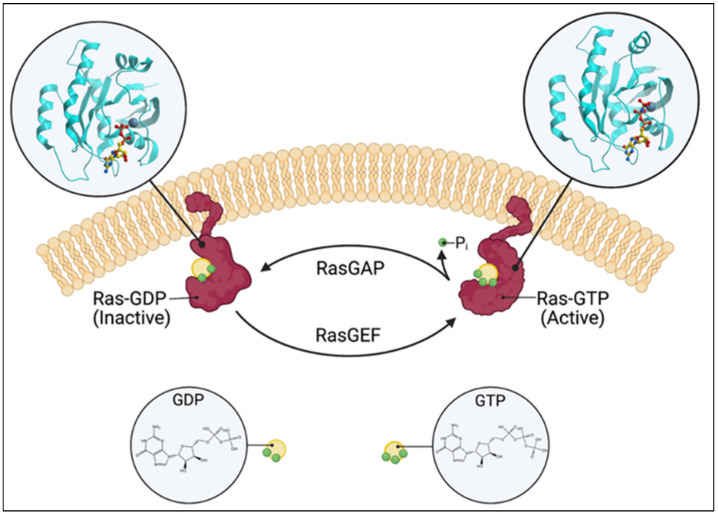
Schematic of the Ras switching cycle. Ras cycles between the GTP-bound active state and the GDP-bound inactive state. RasGAP catalyzes the hydrolysis of GTP, and RasGEF facilitates guanine nucleotide exchange. Created with BioRender.com.

Ras guanine nucleotide exchange factors (RasGEFs) are composed of three families of proteins: Ras guanine nucleotide-releasing proteins (RasGRPs), Son of Sevenless (SOS), and Ras guanine nucleotide-releasing factors (RasGRFs). The RasGRP family consists of four members, RasGRP1, RasGRP2, RasGRP3, and RasGRP4, the SOS family is composed of two members, SOS1 and SOS2, and the RasGRF family is also composed of two members, RasGRF1 and RasGRF2. The commonality is that they catalyze the removal of GDP from GTPases, such as Ras and Rap, and allow for its replacement [1] (Figure 1). While Ras itself possesses intrinsic GTPase and guanine nucleotide exchange activities, the basal activity is low. The activation of the canonical Ras pathway is characterized by the phosphorylation of Raf, Mek, and Erk. The active GTP-bound Ras has a wide range of downstream effects at the cellular level, such as a proliferation, differentiation, and apoptosis. Given these fundamental roles, numerous disease processes have been attributed to the dysregulation of Ras and RasGEFs, which range from autoimmune and inflammatory diseases to neoplasia. A broad review of all RasGEFs in various cell types is beyond the scope of this focused review of RasGRP1 in cancer; however, we direct the reader to previous reviews [2,3,4,5,6]. The structure, function, and regulation of RasGRP1 are briefly discussed, and the role of RasGRP1 in leukemia, lymphoma, squamous cell carcinoma, colorectal cancer, hepatocellular carcinoma, and breast cancer are reviewed in-depth below. 

## 2. RasGRP1: Structure and Function

**Figure 2:**
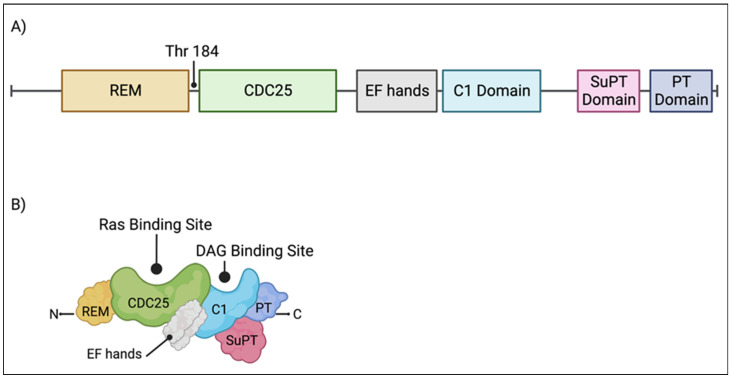
RasGRP1 domains. (**A**) Individual protein domains and the PKC-phosphorylation site, threonine 184, are diagramed and labelled. (**B**) An illustration of RasGRP1 protein domains. The Ras and DAG binding sites are labelled. Created with BioRender.com.

The catalytic module of RasGRP1 is composed of the Ras exchange motif (REM) followed by the CDC25 subunit (Figure 2). Upon the binding of the Ras to the catalytic module of RasGRP1, the helical hairpin of CDC25 removes the GDP from the GTPase. As the cellular concentration of GTP is about 10-fold higher than GDP, GTP occupies the free nucleotide-binding pocket of the enzyme. This hairpin is highly conserved in all GEFs [7]. A single amino acid change within this structure has been shown to abrogate its catalytic effect [8,9]. Despite the ability of the helical hairpin alone to convey the nucleotide dissociating ability of RasGEFs, the nucleotide exchange step is mediated by other regions of the catalytic domain [10].

Adjacent to the catalytic module of RasGRP1 is a pair of EF hands (Figure 2) with a calcium-binding capacity in vitro [11]. The evidence is conflicted on the importance of EF hands and calcium for the activation of RasGRP1. Some studies found the EF hands and calcium to be dispensable [12,13,14], while some found them to be necessary [15]. The current weight of evidence supports the idea that the EF hands do indeed bind calcium, induce conformational changes, and activate RasGRP1 [16,17]. The calcium source is from the endoplasmic reticulum stores, and its release is mediated by phospholipase C-γ-generated inositol-1,4,5-trisphosphate (IP3) [18]. 

The C1 domain of RasGRP1 binds DAG (Figure 2), generated by PLCγ, and its synthetic analog phorbol myristate acetate (PMA) and 12-O-tetradecanoylphorbol-13-acetate (TPA) [11,19,20,21]. Upon the binding of the C1 domain to the DAG, RasGRP1 becomes anchored to the plasma membrane [11,13,22,23,24,25,26] where its substrate, GDP-loaded GTPase, is present. Alternatively, RasGRP1 can also be trafficked to the endoplasmic reticulum (ER) and Golgi apparatus [23,24,27,28,29]. While the ability of the C1 domain to bind DAG is well known, it alone is insufficient for membrane targeting and requires other domains on RasGRP1, which are discussed below. 

The tail region of RasGRP1 possesses an approximately 140 residue-long coiled-coil (CC), later renamed as the plasma membrane-targeting (PT) domain, and the suppressor of PT (SuPT) domain [16,23] (Figure 2). In the inactive state, the SuPT domain of RasGRP1 attenuates the plasma membrane-targeting activity of the PT domain [23]. Upon the binding of the C1 domain to DAG, it also counteracts the SuPT domain and enables the PT domain to target RasGRP1 to the plasma membrane [23,30]. At the plasma membrane, the hydrophobic residues of the PT domain bind phospholipid vesicles containing phosphoinositides. The deletion of the hydrophobic residues prevents the PI3k-dependent plasma membrane targeting of RasGRP1 [30], and the deletion of the tail region entirely leads to a T cell dysregulation [31]. The PT domain additionally facilitates the dimerization of RasGRP1 in the inactive state [16]. While the C1 domain is well-recognized for its role mediating the RasGRP1 membrane targeting capacity and activation, it is now accepted that these effects are also dependent on the tail domain of RasGRP1.

## 3. Ras Guanine Nucleotide-Releasing Protein 1: Regulation

**Figure 3:**
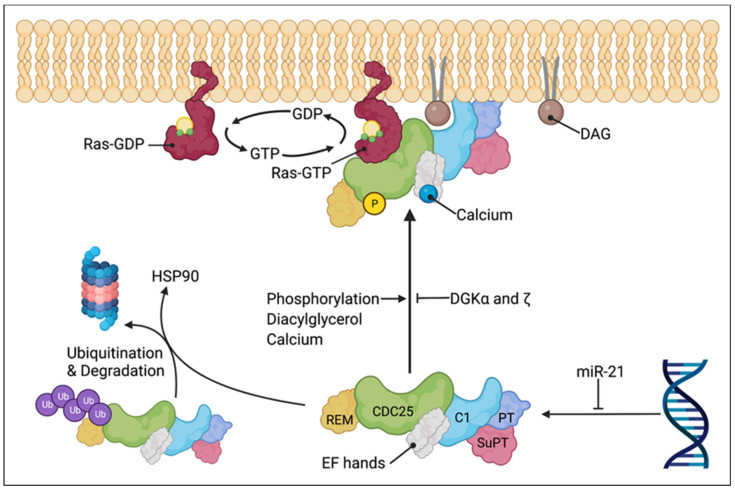
Model of RasGRP1 regulation and plasma membrane translocation. RasGRP1 is sequestered in the cytoplasm in its inactive state (lower center) and translocated to the plasma membrane or endomembrane upon activation (upper center). RasGRP1 activation involves phosphorylation of threonine 184, DAG binding by C1 domain, and calcium binding by the EF hands. Termination of RasGRP1 signaling is mediated by the breakdown of DAG to PA by DGKα and DGKξ. RasGRP1 can be suppressed by miR-21 and degraded following disassociation from HSP90 and polyubiquitination. Active RasGRP1 catalyzes the release of GDP from Ras and facilitates their transition from inactive GDP-bound to active GTP-bound state. Created with BioRender.com.

The translocation and activation of the RasGRP1 membrane are reliant on the binding of the C1 domain to DAG (Figure 3). Logically, the catalyzation of DAG to phosphatidic acid (PA) by diacylglycerol kinases (DGK) should terminate RasGRP1 signaling. Indeed, DGKα is recruited to the plasma membrane after the TCR stimulation [32] and results in suppressed RasGRP1 activity and Ras signaling [33,34]. This mechanism of RasGRP1 regulation has been proposed to be a mechanism of T cell anergy [35,36]. Other DGK isoforms also regulate the RasGRP1 activity, specifically, DGKζ. Studies have found that the overexpression of kinase-dead DGKζ in Jurkat cells prolonged the Ras activation, and the overexpression of the wild-type DGKζ suppressed the ERK phosphorylation following the TCR ligation [37,38]. For in-depth reviews of the DGKs, we direct the reader to previous reviews [39,40].

The activity of RasGRP1 is regulated by multiple mechanisms in addition to endomembrane versus plasma membrane localization. RasGRP1 is also regulated by the phosphorylation (Figure 3), more specifically, of threonine 184 (T184) by protein kinase C α (PKCα) after the TCR engagement or PMA stimulation [41]. While the phosphorylation of T184 enhances the activity of RasGRP1, it is not completely required. A RasGRP1 Thr184Ala mutant did not exhibit a significant signaling defect [42]. DGKζ not only indirectly regulates the RasGRP1 activity via DAG, but it also physically associates with PKCα and inhibits the phosphorylation of RasGRP1 [43]. In unstimulated cells, RasGRP1 is believed to exist in an autoinhibited dimeric form, in which the EF domains of each monomer block DAG-binding sites on the C1 domain of the partner. It was also suggested that an invariant His 212 in RasGRP1, 2, and 3 functions as a pH sensor: lymphocyte receptor stimulation causes an increase in the intracellular pH and thus the deprotonation of His 212 [44]. The later causes the structural rearrangement of the linker between the CDC25 and EF domain and the destabilization of the autoinhibition [44]. We refer the reader to the review by Griner and Kazanietz for additional details on PKC and other DAG effectors [45].

Ding and colleagues identified RasGRP1 to be a client protein of the chaperone heat shock protein 90 (HSP90) (Figure 3). Additionally, the degradation of RasGRP1 can be mediated by HSP90 acetylation [46]. There is emerging evidence that microRNAs also play a role in the RasGRP1 expression; specifically, miR-21 was shown to suppress the expression of RasGRP1 [47,48]. Conversely, the downregulation of miR-21 increased the RasGRP1 expression in vitro [49]. 

## 4. RasGRP1: Cell Development and Function

### 4.1. Immature Thymocytes 

**Figure 4:**
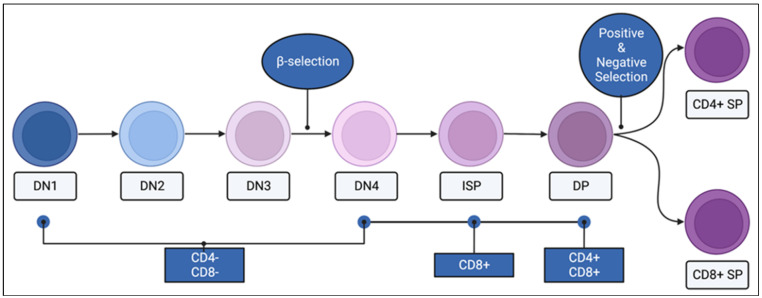
CD4 and CD8 expression of thymocytes through maturation. Immature thymocytes begin the process of maturation in the thymus in the double negative (DN; CD4− CD8−) stage, followed by DN2, DN3, and DN4 stages. The process of β-selection takes place as DN3 thymocytes mature into the DN4 stage. In mice, DN4 thymocytes transition through an immature single positive (ISP, CD8+) stage to become double positive (DP; CD4+ CD8+) thymocytes. The second round of selection, positive and negative selection, takes place between the DP and the mature single positive (SP) stages. Mature SP thymocytes can be CD4+ or CD8+. Created with BioRender.com.

The maturation of immature thymocytes undergo four double-negative (CD4−, CD8−) stages (DN1–4), an immature single positive stage (ISP; CD8+ in mice and CD4+ in humans), and a double positive (DP; CD4+, CD8+) stage (Figure 4). During this process, immature thymocytes undergo two selection checkpoints. The first of which is a process termed “β-selection”, which takes place in the DN3-DN4 transition stage, where the pre-T cell receptor (pre-TCR), composed of a somatically rearranged TCRβ chain and an invariant pre-TCRα chain, signal a first intersect with the Ras pathway. This signal is necessary for the αβ T cell precursors to eventually become mature CD4+ or CD8+ T cells. The activation of the Ras is critical in β-selection; in fact, activated Ras can replace the pre-TCR expression and generate DP thymocytes in *Rag*^−/−^ mice [50]. At the β-selection step, RasGRP1 is dispensable and acts as a backup for SOS1 [51,52,53]. Immature thymocytes that pass the β-selection undergo a proliferative burst and initiate CD4 and CD8 expression to become DP thymocytes. DP thymocytes that express TCRαβ undergo subsequent checkpoints termed “positive” and “negative” selection, where the TCRαβ signal quality and strength are interrogated. RasGRP1 plays an essential role in a positive selection, and SOS1 acts as backup in a negative selection [52,53]. The knockout of RasGRP1 arrests the progression of DP thymocytes through a positive selection [51,54], whereas the double knockout of RasGRP1 and SOS1 is needed to arrest a negative selection [53]. 

**Figure 5:**
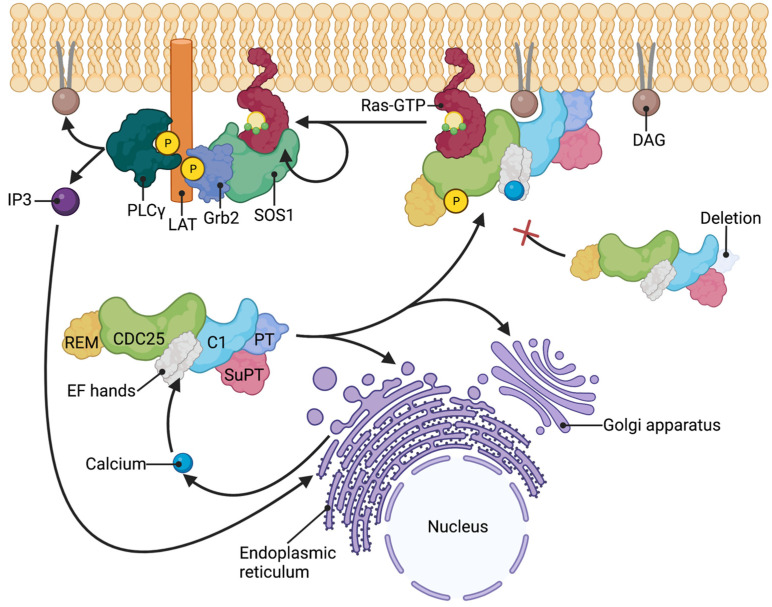
Model of RasGRP1 membrane translocation. RasGRP1 activation is characterized by phosphorylation of threonine 184, binding of calcium by the EF hands, and translocation to the plasma membrane or endomembranes (Golgi apparatus and endoplasmic reticulum). Deletion of the PT domain renders RasGRP1 unable to translocate to the plasma membrane. In negative selection of αβTCR-expressing thymocytes, RasGRP1 is recruited to the plasma membrane along with SOS1-Grb2 complex. Grb2-SOS1 complex binds phospho-LAT and RasGTP at the plasma membrane and act as another RasGEF. PLCγ is also recruited to the phospho-LAT at the plasma membrane during RasGRP1 activation and generates DAG and IP3. IP3 subsequently induces the release of intracellular stores of calcium. Created with BioRender.com.

The compartmentalization of Ras signaling underlies the digital output (positive versus negative) seen in the pre-TCR and TCR selection checkpoints [55,56,57] (Figure 5). Negative selection signaling is molecularly characterized by the plasma membrane recruitment of the RasGRP1 and Grb2 (growth factor receptor-bound protein 2)-SOS1 complex from the cytosol, and the resultant activation of the Ras pathway [55,56,57]. The Grb2-SOS1 complex binds phosphorylated LAT at the plasma membrane and serves as another RasGEF. However, positive selecting signaling is characterized by the recruitment of RasGRP1 to the Golgi apparatus, and no involvement of the Grb2-SOS1 complex [55,56].

### 4.2. T Cells

Despite the maturation arrest of thymocytes and the loss of mature single-positive thymocytes in RasGRP1 knockout mice, this arrest is not complete. RasGRP1-deficient CD4+ and CD8+ T cells do exist [58], however, they are defective in their capacity to become activated and proliferate after an anti-CD3 and anti-CD28 antibody stimulation [58]. Interestingly, humans deficient in RasGRP1 have increased numbers of TCRγδ+ CD8+ T cells [58]. RasGRP1-deficient mice develop splenomegaly and autoantibodies as a result of T cell dysregulation, characterized by an elevated interleukin (IL)-4 secretion [31,59,60,61]. This elevation in IL-4 drives B cell proliferation and the production of autoantibodies [60]. Furthermore, one study found that the binding of RUNX1 to a putative autoimmunity-associated enhancer 1 upstream of *Rasgrp1* mediates the RasGRP1 deficiency-mediated autoimmune disease [61].

### 4.3. B-Cells

B cells express RasGRP1 and RasGRP3. While both are involved in B cell receptor (BCR)-mediated Ras signaling, RasGRP3 plays the central role [59,62]. One study found that the BCR-mediated proliferation was suppressed more by the knockout of RasGRP3 than RasGRP1 and was absent in double knockouts [59]. The defect in the B cell proliferation due to the RasGRP1 knockout was supported by a later study [58]. Unlike T cells, the knockout of both RasGRP1 and RasGRP3 did not disrupt the development of B cells [59]. However, B cells that express a dominant negative Ras mutant have severe developmental defects at the pre–pro B cell stage [63,64]. 

### 4.4. NK Cells

NK cells exert their cytotoxic effect and produce cytokines and chemokines subsequent to the activation of various cell surface receptors [65]. Briefly, this signal cascade is dependent on RasGRP1, and the knockdown of RasGRP1 in NK cells results in a markedly decreased cytokine production and cytotoxicity [58,66]. In humans, this defect has been attributed to the protein–protein interaction between RasGRP1 and the dynein light chain (*Dynll1*) [58].

### 4.5. Granulocytes

The differentiation of myeloid progenitors into neutrophils is dependent on the transcription factor growth factor independence 1 (Gfi1) [67,68,69] and the growth factor granulocyte colony-stimulating factor (G-CSF) [70,71,72]. Gfi1 regulates G-CSFR signaling in myeloid progenitors via the upregulation of the RasGRP1 expression and Ras activation [73]. RasGRP1 has also been found to be important for a mast cell degranulation. RasGRP1^−/−^ mice exhibit an impaired immunoglobulin E (IgE)-mediated degranulation and anaphylaxis [74].

## 5. RasGRP1: Role in Cancer

### 5.1. Lymphoma and Leukemia

While loss-of-function RasGRP1 mutants have been described in humans [75,76,77], no oncogenic mutant of RasGRP1 has been identified. These loss-of-function RasGRP1 mutants lead to the development of autoimmune lymphoproliferative syndrome (ALPS), CD4+ T cell lymphopenia, recurrent infections, hepatosplenomegaly, and lymphadenopathy [75,76,77]. It is important to note that some patients with loss-of-function RasGRP1 mutants develop Epstein–Barr virus (EBV)-induced B cell lymphoma. However, studies have found RasGRP1 to be overexpressed in nearly half of all T cell acute lymphoblastic leukemias (T-ALL) [78,79]. Retroviral insertion studies in mice have also identified wild-type RasGRP1 as a leukemogenic oncogene [80,81,82]. Furthermore, the dysregulation of RasGRP1 in mice and cell lines has been shown to lead to the development of thymic lymphomas and T cell leukemias [79,83,84]. Interestingly, cell lines with a high RasGRP1 expression required a cocktail of IL-2, -7, and -9 for proliferation [79,84]. Additionally, leukemia driven by the overexpression of RasGRP1 and *K-Ras^G12D^* are mutually exclusive and represent the distinct mechanisms of leukemogenesis [79]. This is consistent with the finding from a later study that identified RasGRP1 as a negative regulator of Ras signaling in Kras^−/−^ Nras^Q61R/+^-driven leukemia [85]. Various studies have shown that the dysregulation of RasGRP1 itself is insufficient for leukemogenesis [79,86]; however, it does bestow a proliferative advantage in bone marrow progenitors over wild type cells [86]. Consistent with Knudson’s “two-hit” theory that was proposed over 50 years ago [87], the dysregulation of RasGRP1 requires a second cooperating oncogene or cytokine stimulation for transformation [78,79,84]. The knockout of RasGRP1 negative regulators has also been shown to be oncogenic; specifically, DGKα^−/−^ DGKζ^−/−^ double knockout mice develop thymic lymphoma due to the failure to prevent the overactivation of RasGRP1 and Ras [88]. Beyond the role of RasGRP1 as an oncogene, its overexpression has been documented to be a mechanism of resistance to MEK inhibitors [89].

No RasGRP1-specific small molecule inhibitors currently exist. Since the overexpression of RasGRP1 renders T-ALL cells responsive to pro-tumorigenic cytokines [84], PI3K inhibitors have been tested as a monotherapy in mice, but with no success [90]. Others have tried to target the RasGRP1/Ras/Erk pathway in T cell lymphoblastic lymphomas (T-LBL), which are morphologically and immunophenotypically identical to T-ALL [91]. Bromodomain-containing protein 2 (BRD2) binds to the promotor region of *Rasgrp1* and conveys a doxorubicin resistance in some T-LBL patients [92]. The targeting of BRD2 via a bromodomain and extra-terminal (BET) inhibitor improved the therapeutic efficacy in vitro and in a patient-derived xenograft mouse model [92]. DAG and its analogues have long been known to activate RasGRP1 in T and B cells [93,94], and the treatment of B cell lymphoma-derived cell lines with DAG analogues promoted apoptosis [94,95]. This proapoptotic pathway induced by DAG analogues is mediated by the PKC/RasGRP1/Erk pathway [94,95].

### 5.2. Squamous Cell Carcinoma

While studying the role of RasGRP1 in skin tumors, one group found that the overexpression of RasGRP1, driven by a K5 promotor, in keratinocytes resulted in the development of spontaneous skin tumors [96,97]. These tumors were mostly benign papillomas and there were lesser numbers of squamous cell carcinomas. Due to the observation that the incidence of tumors development was higher in co-housed animals, it was hypothesized that wounding contributed to tumor development. Indeed, when RasGRP1-K5 transgenic mice were subjected to full-thickness incision wounding, 50% of them developed skin tumors [97]. The proposed mechanism is that the act of wounding caused the release of the granulocyte colony-stimulating factor (G-CSF) by keratinocytes [96,97], and G-CSF acted in an autocrine and paracrine fashion to cooperate with RasGRP1 in the development of skin tumors [98]. When the same RasGRP1-K5 transgenic mice were subjected to multistage skin carcinogenesis protocol, 7,12-dimethylbenz(a)anthracene (DBMA) as carcinogen, and 12-O-tetradecanoylphorbol-13-acetate (TPA) as tumor promoters, it was found that the squamous cell carcinomas that developed in the transgenic mice were larger, less differentiated, and more invasive [99]. Additionally, the overexpression of RasGRP1 was found to partially replace the DMBA induction [99]. Conversely, RasGRP1 knockout mice have impaired skin tumorigenesis, evidenced by a reduced epidermal hyperplasia induced by TPA [100,101]. To study other coopering mechanisms of oncogenesis in keratinocytes, one group transduced keratinocytes derived from a Li-Fraumeni patient with RasGRP1 and found that the keratinocytes acquired morphologic changes that are associated with a transformation [102]. This result supports the idea that RasGRP1 cooperates with other genes because patients with Li-Fraumeni syndrome are deficient in p53, a well-known tumor suppressor gene.

### 5.3. Colorectal Cancer

Surprisingly, RasGRP1 acts as a tumor suppressor in colonic epithelium; furthermore, RasGRP1 can be used as a biomarker for predicting the efficacy of anti-epidermal growth factor receptor (EGFR) therapy for CRC (colorectal cancer) patients [103,104]. The RasGRP1 expression levels decrease with the progression of CRC and predict the poor clinical outcome of patients [104]. Mechanistically, the same group found that RasGRP1 suppresses the proliferation of the KRas mutant and negatively regulates the EGFR/SOS1/Ras signal in CRC cells [104]. This mechanism may explain its tumor suppressor activity in colorectal cancer in contrast to its oncogenic activity in most other neoplasias.

### 5.4. Hepatocellular Carcinoma

RasGRP1 has been found to be upregulated in hepatocellular carcinomas (HCC) [105]; furthermore, a high RasGRP1 expression is associated with the tumor size, tumor–node–metastasis (TNM) stage, and Barcelona Clinic Liver Cancer stage [105]. At the cellular level, in Huh7 and PLC cells, the downregulation of RasGRP1 inhibited cell proliferation, whereas the overexpression of RasGRP1 promoted cell proliferation [105]. Specific protein 1 (Sp1) was identified to bind the *Rasgrp1* promotor and is a positive regulator [105]. For a review of the Ras pathways in HCC, we refer the reader to the work by Moon and colleagues [106].

### 5.5. Breast Cancer

The role of RasGRP1 in breast cancer has only recently been studied. Specifically, it was found that the upregulation of *Rasgrp1* was associated with an improved overall survival in breast cancer [107], as well as overall survival and disease-free survival in the triple-negative breast cancer subtype [107,108]. The molecular mechanism that underlies these observations is unknown.

## 6. Conclusions

Given that approximately 46% of cancers exhibit alterations in the Ras pathway [109], it has been extensively studied over the past decades. With RasGRP1 being a RasGEF, it too has received much attention. Through this endeavor, the structure, function, regulation, and developmental role of RasGRP1 have been described at the molecular level. This has identified RasGRP1 and its regulators as promising targets in leukemia and other cancers.

Most of the domains of RasGRP1 are well characterized. The REM and CDC25 domains facilitate the Ras cycle between the GDP-bound inactive form and the GTP-bound active form. The EF hands bind calcium and induce an activation-associated conformational change [16,17]. The C1 domain binds DAG at the plasma membrane or endomembrane. The PT domain facilitates dimerization and phosphoinositide-mediated plasma membrane targeting [30]. For the regulation of RasGRP1, it is known that signal termination can be mediated by DGKα and DGKζ via the conversion of DAG to PA. For the activation, RasGRP1 can be phosphorylated at T184 by PKCα. Other less-well characterized mechanisms include HSP90- [46] and miR-21-mediated degradation [47,48].

In normal physiology, RasGRP1 plays an important role in the maturation of thymocytes. Specifically, it is necessary for a positive selection of the rearranged αβTCR [52,53]. The compartmentalization of Ras signaling to the plasma membrane or the endomembrane at the selection checkpoints adds an extra layer of complexity [55,56,57]. The dysregulation of RasGRP1 in peripheral T cells, B cells, NK cells, neutrophils, and mast cells are known to cause developmental and/or functional defects. One of the most surprising defects revealed in knockout mice is that RasGRP1 normally interacts with the dynein light chain in NK cells [58], and this indicates that RasGRP1 has additional functions besides as a RasGEF.

Given the importance of RasGRP1 in cell development, it is unsurprising that it is expressed in numerous cancers and plays a role in oncogenesis. The overexpression of RasGRP1 alone is insufficient for lymphoma- or leukemo-genesis [79,86]. The transformation of thymocytes requires the overexpression of RasGRP1 and a cooperating oncogene or knockout of a tumor suppressor. Since no Ras- or RasGRP-specific small molecule inhibitors have been identified, efforts have been made to target regulatory pathways through the use of BET inhibitors [92], DAG analogs [94,95], and HDAC inhibitors [46].

Much of the work done on RasGRP1 within the realms of immunology and cancer research in the last 5 years has focused on three areas. The first area is its role in lymphocyte homeostasis, which can be summarized by the identification of loss-of-function RasGRP1 mutants in two patients with ALPS [75], one patient with immunodeficiency, and three patients with EBV-associated lymphoproliferative disease [76,77,110]. A second area is the clinical behavior of tumors relative to the expression of RasGRP1 in various cancers, such as CRC [103], HCC [105], and breast cancer [107,108]. The third area is the mechanism by which RasGRP1 serves as a tumor suppressor in certain cancer models [85,111]. These last two emerging areas point to the idea that RasGRP1 cannot simply be described as an “oncogene” or its overexpression as a negative indicator, but rather that its role is cancer- and model-dependent. While not emphasized in this focused review, progress in RasGRP1 research is also being made in the areas of schizophrenia [112], neuro-inflammation [113], systemic lupus erythematosus [114], Parkinson’s disease [115], and angiogenesis [116]. It is evident that the relevance of RasGRP1 reaches beyond the development and function of immune cells and homeostasis and cancer.

Despite this progress, there is still much to understand about RasGRP1. First, a concise explanation for the conflicting role of calcium, or lack of, in the function of RasGRP1 has yet to be articulated. Second, since RasGRP1 is involved in the degranulation of NK cells and mast cells and the development of neutrophils, it is interesting to speculate on its potential developmental and functional role in other granulocytes. It is clear that RasGRP1 plays a role in T leukemogenesis; additionally, it is necessary for it to cooperate with other oncogenes for transformation. It is likely that the array of cooperating oncogenes has yet to be fully elucidated. Lastly, only in recent years was RasGRP1 identified as a differentially expressed gene correlated with overall and disease-free survival in breast cancer. It will be important to determine the molecular basis for this counterintuitive correlation.

## Abbreviations

12-O-tetradecanoylphorbol-13-acetate (TPA), 7,12-dimethylbenz(a)anthracene (DBMA), autoimmune lymphoproliferative syndrome (ALPS), B cell receptors (BCR), bromodomain and extra-terminal (BET), bromodomain-containing protein 2 (BRD2), coiled-coil (CC), colorectal cancer (CRC), diacylglycerol (DAG), diacylglycerol kinase (DGK), endoplasmic reticulum (ER), epidermal growth factor receptor (EGFR), Epstein–Barr virus (EBV), granulocyte colony-stimulating factor (G-CSF), growth factor independence 1 (Gfi1), growth factor receptor-bound protein 2 (Grb2), guanine nucleotide exchange factors (GEFs), guanosine diphosphate (GDP), guanosine triphosphate (GTP), heat shock protein 90 (HSP90), hepatocellular carcinomas (HCC), immunoglobulin E (IgE), inositol-1,4,5-trisphosphate (IP3), interleukin (IL), natural killer cells (NK cells), phorbol myristate acetate (PMA), phosphatidic acid (PA), phospholipase Cγ (PLCγ), plasma membrane-targeting (PT), pre-T cell receptor (pre-TCR), protein kinase C (PKC), Ras exchange motif (REM), Ras exchange motif (REM), Ras guanine nucleotide-releasing factors (RasGRFs), Ras guanine nucleotide-releasing protein 1 (RasGRP1), runt-related transcription factor 1 (RUNX1), Son of Sevenless (SOS), specific protein 1 (Sp1), suppressor of PT (SuPT), T cell acute lymphoblastic leukemias (T-ALL), T cell lymphoblastic lymphomas (T-LBL), and T cell receptor (TCR).
